# Long non-coding RNAs potentially suppressive for brain perivascular astrogliosis induced by environmental ultrafine particulate matter

**DOI:** 10.1007/s11356-025-37141-5

**Published:** 2025-10-31

**Authors:** Hiyori Edo, Ryodai Itano, Masakazu Umezawa

**Affiliations:** 1 Department of Materials Science and Technology, Graduate School of Advanced Engineering, Tokyo University of Science, 6-3-1 Niijuku, Katsushika, Tokyo, 125-8585 Japan; 2 Department of Medical and Robotic Engineering Design, Faculty of Advanced Engineering, Tokyo University of Science, 6-3-1 Niijuku, Katsushika, Tokyo, 125-8585 Japan

**Keywords:** Perivascular astrogliosis, LncRNA, Environmental particulate matter, Mice, Neurovascular disorders, Brain cerebral cortex

## Abstract

Respiratory exposure to low doses of environmental ultrafine particulate matter induces perivascular astrogliosis lesions in the cerebral cortex, associated with neurodegenerative and neurodevelopmental disorders. Proteins with abnormal secondary structures accumulate in such lesions during conditions such as dementia. In addition to the upregulation of glial fibrillary acidic protein (*Gfap*) and aquaporin 4 (*Aqp4*) genes involved in pathogenesis, several long non-coding RNAs (lncRNAs) are differentially expressed in the lesion; however, their functions remain unclear. This study aimed to explore the potential roles of lncRNAs in suppressing perivascular lesions. RNA expression profiles of the cerebral cortex of 6-week-old mice with perivascular lesions were analyzed using principal component analysis to identify lncRNAs whose expression was inversely correlated with the lesion severity. Common sequences among these lncRNA groups and associated mRNAs with their complementary regions were identified. Identified mRNAs were analyzed using the DAVID functional enrichment tool. A shared sequence, “AUCAUCUUUCC,” was found in potentially lesion-suppressive lncRNAs inversely correlated with lesion extent. The complementary mRNAs were enriched in eight Gene Ontology terms, including “Cell projection,” “Dendrites,” “Synapses,” and “Projection neurons,” related to astrocytic endfoot elongation observed in the perivascular lesions and in neural functions in the brain. The identified lncRNAs and their common sequences may represent novel regulators of cellular development and lesion dynamics. The potential of RNA molecules with this sequence to modulate the pathogenesis of neurodegenerative and neurodevelopmental disorders warrants further investigation.

**Graphical Abstract:**

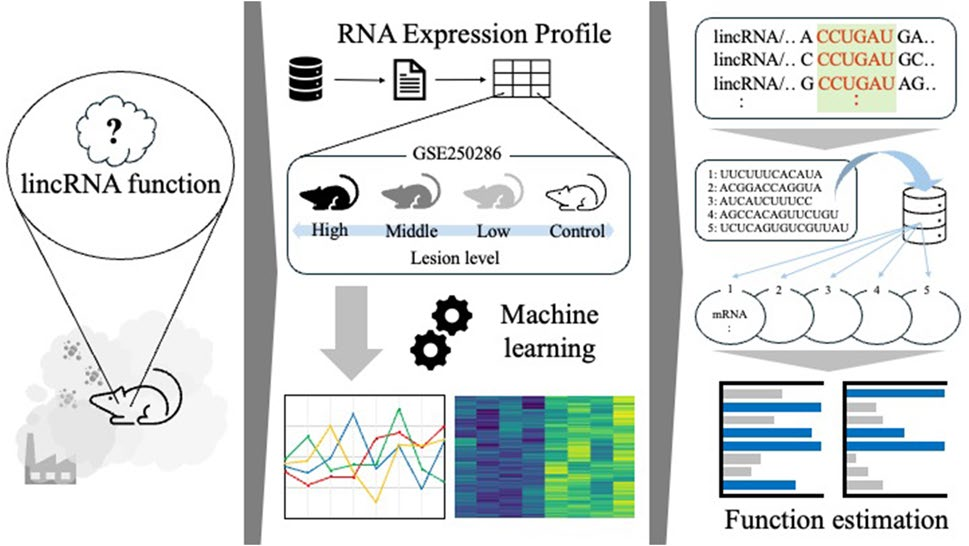

## Introduction

Unintentional exposure to environmental ultrafine particulate matter affects brain function (Brunekreef and Holgate 2002; Hu and Gao 2010; Power et al. 2011; Kioumourtzoglou et al. 2016; Zhao et al. 2020b). In particular, perivascular cells in the brain are sensitively and persistently affected by exposure to a low dose of carbon black nanoparticles during the fetal period. This exposure induces a pathology characterized by increased accumulation of β-sheet structure (Onoda et al. 2017a) with sustained endoplasmic reticulum stress, and degeneration of brain perivascular macrophages (Onoda et al. 2014, 2017c; Umezawa et al. 2018) with abnormal neurobehavior (Umezawa et al. 2018). These lesions can be suppressed by pretreatment with an antioxidant, N-acetyl cysteine (Onoda et al. 2017b).

The increase in the perivascular protein β-sheet structures in the brain resembles a pathological finding of various neurodevelopmental disorders involving the accumulation of abnormal protein aggregation in the brain. Alzheimer’s disease, a major neurodegenerative disease, is associated with such aberrant structural protein accumulation of amyloid β and tau (Jacobsen et al. 2006; Fu et al. 2018). Thus, a close association is suspected, and cerebral perivascular lesions may increase the risk of neurodegenerative disorders as well as neurodevelopmental disorders with abnormal neurobehaviors such as autistic spectrum disorder. Increased expression of glial fibrillary acidic protein (*Gfap*) and aquaporin 4 (*Aqp4*) (Onoda et al. 2017b) has been observed in the perivascular lesions following maternal exposure to inorganic nanoparticles. While Gfap constitutes the astrocyte cytoskeleton and is a key molecular marker for astrocyte activation and reactive astrogliosis, Aqp4 is a water-selective channel highly expressed in the perivascular membrane of reactive astrocytic endfeet (Onoda et al. 2017c). These RNAs have been implicated in the pathogenesis of the perivascular lesions.

RNA transcribed from DNA is broadly classified into mRNA, which encodes proteins, and non-coding RNA (ncRNA), which does not. ncRNAs include microRNAs (miRNAs), approximately 20–25 nucleotides long, and long non-coding RNAs (lncRNAs), typically over 200 nucleotides. LncRNAs are transcribed by RNA polymerase II from various genomic regions, including introns, intergenic regions, enhancers, and promoters (Novikova et al. 2013; García-Fonseca et al. 2021), comprising the non-coding genome that accounts for over 98% of human DNA. Approximately 28,000 lncRNA species have been identified (Novikova et al. 2013; Iwakiri 2022). These molecules regulate gene expression through diverse mechanisms; for example, by interacting with mRNA to control gene expression, and are classified as decoys, guides, scaffolds, or precursors (García-Fonseca et al. 2021). As decoys, lncRNAs bind to target miRNAs and prevent their functions. As guides, lncRNAs recruit proteins involved in epigenetic regulation, thereby controlling gene expression. As scaffolds, lncRNAs form a complex with effector proteins to bind them to the DNA-binding sites of specific genes. In addition, as precursors, lncRNAs can be converted to miRNAs that regulate gene expression (García-Fonseca et al. 2021).

LncRNAs regulate gene expression through complex interactions with proteins, miRNAs, and mRNAs by functioning as competing endogenous RNAs (Tay et al. 2014). Several lncRNAs have been implicated in various diseases including cancer (Tay et al. 2014), fatty liver disease (Zhao et al. 2018b), dementia (Li et al. 2019; Yi et al. 2019; Huang et al. 2020; Sen and Mukhopadhyay 2024), and cerebrovascular diseases (Feng et al. 2022). Databases such as “lncRNA and Disease Database” (Chen et al. 2013; Bao et al. 2019) and “LncTarD” (Zhao et al. 2020a) have been developed to investigate the involvement of lncRNAs in various pathological processes. Although many of the functions of lncRNAs remain unknown, their stability in the peripheral circulation and detectability by using qRT-PCR and other methods make them suitable biomarkers for the diagnosis of lung adenocarcinoma (Zhao et al. 2018a), Alzheimer’s disease (Faghihi et al. 2008), and amyotrophic lateral sclerosis (Gagliardi et al. 2018). However, the functional roles of lncRNAs differentially expressed in perivascular lesion remain quite unclear.

Therefore, in this study, we aimed to investigate the functional characteristics of lncRNAs differentially expressed in perivascular lesions and identify the features of RNA molecules that potentially suppress perivascular lesions in the brain. We focused on the potential of lncRNAs, whose expression levels were inversely correlated with the level of perivascular astrogliosis in the brain induced by fetal exposure to carbon black nanoparticles, a model of environmental ultrafine particulate matter. These RNAs may represent novel molecules that suppress perivascular brain lesions linked to neurodegenerative and neurodevelopmental disorders. We also aimed to identify the key sequences of lncRNAs that can potentially suppress lesions by characterizing downregulated lncRNAs and analyzing the functional roles of mRNAs that can bind to these key lncRNA sequences.

## Materials and methods

### Data source and data preprocessing

We used the GSE250286 dataset deposited in the Gene Expression Omnibus database (https://www.ncbi.nlm.nih.gov/geo/). The dataset contains RNA microarray profiles from the cerebral cortex of mice with and without perivascular lesions. Astrogliotic lesions were induced in offspring by maternal exposure to carbon black nanoparticles. This dataset contains eight transcriptomic samples representing different lesion levels (Onoda et al. 2017c). Each sample originally contained 16,251 lncRNAs.

Preprocessing involved filtering quantitative microarray signals. First, lncRNA data were filtered based on the value of gScaleEval (expressed as three levels of confidence: 0, 1, and 2, with 2 representing the gene expression intensity with the highest confidence in the quantification). Genes were retained only if at least one of the eight samples had a gScaleEval score of 2. Non-lncRNAs were excluded. For lncRNAs, the differences in expression levels between samples were calculated for each gene under the same conditions. The mean value and standard deviation of the obtained expression differences were calculated across all genes, and genes that did not fall within the standard deviation during all three replicates were considered outliers and excluded from the analysis. Finally, weakly expressed genes with expression intensity less than 100 in any sample were also excluded. After preprocessing, 12,135 lncRNAs were included in the analysis.

### Standardization of data

*Z*-score standardization was used to normalize gene expression values. The formula for *Z*-score standardization is shown below, where the gene expression intensity in each sample is ${\text{I}}_{\text{n}}$(*n* = 1–8), the mean of gene expression intensity for each gene is $\overline{{\text{I} }_{\text{n}}}$, the standard deviation is *σ*, and the standardized value is ${\text{x}}_{\text{n}}$(*n* = 1–8).

1 $$x_n=\frac{I_n-\overline{I_n}}\sigma$$

### Principal component analysis to extract lncRNAs inversely correlated with the lesion extent

Principal component analysis (PCA), an unsupervised machine-learning model, was used to aggregate high-dimensional data with many variables in the form of principal components. Visualizing high-dimensional data structures is challenging; however, using PCA, the data can be aggregated to a lower dimension, and the structure of the data can be visualized. In this study, PCA helped identify lncRNAs inversely correlated with lesion severity and potentially involved in lesion suppression.

### Extraction of enriched sequences in lncRNAs downregulated in the brain perivascular lesion

To avoid including coincidental sequence matches, common sequences were extracted under the following conditions: at least 10% or more common nucleic acids (at least 10 nucleotides long) were located at the same position in the base sequences of downregulated lncRNAs. In addition, although the defined condition was determined based on the numerical value of the consensus, there were cases in which “+” was displayed instead of a numerical value. The notation indicated that two or more bases occupy the same proportion of the same position in each lncRNA sequence but were not included in the extracted sequence. The results obtained by Clustal Omega were displayed using Jalview, a free program for editing, visualization, and analysis of multiple sequence alignments (Sievers et al. 2011).

### Search for mRNAs with complementary sequences of common lncRNA sequences involved in lesion suppression and confirmation of their expression in the cerebral cortex

Using Basic Local Alignment Search Tool (BLAST), mRNAs with complementary sequences of common lncRNA sequences involved in suppressing lesions were extracted. The following parameters were applied.

- Database (in Choose Search Set): Reference RNA sequences (refseq_rna)
- Organism: Mus musculus (taxid: 10,090)
- Max target sequences (in General Parameters in the Algorithm parameter column): “5000”
- Filter (in Filters and Masking): Species-specific repeats for Mus musculus (House mouse)

Sequences labeled with names other than mRNA such as “PREDICTED” and “cDNA” in “Description” were excluded; mRNAs with a common complementary sequence were extracted if the sequence direction matched that of the searched sequence from “Alignments” information, and the sequence match rate against the searched sequence was at least 85%. Expression of the resulting mRNAs in the cerebral cortex was verified using The Human Protein Atlas, considering expression in “Tissue RNA - cerebral cortex” or “Brain RNA - cerebral cortex” as confirmation (Lindskog 2016).

### Statistical functional analysis of extracted mRNA groups using DAVID

For each common lncRNA sequence involved in lesion suppression in the cerebral cortex, a list of corresponding mRNAs with common and complementary sequences was generated. Functional enrichment analysis was performed using the Database for Annotation, Visualization, and Integrated Discovery (DAVID) to identify overrepresented biological processes (Dennis G Jr et al. 2003). The following parameters were used: “fold enrichment ≥ 2.0” and “false discovery data (FDR) ≤ 0.05.” Functions that met these conditions were considered statistically significantly enriched within the mRNA group.

## Results

### Extraction of lncRNAs involved in the suppression of perivascular lesions in the brain by PCA

**Fig. 1:**
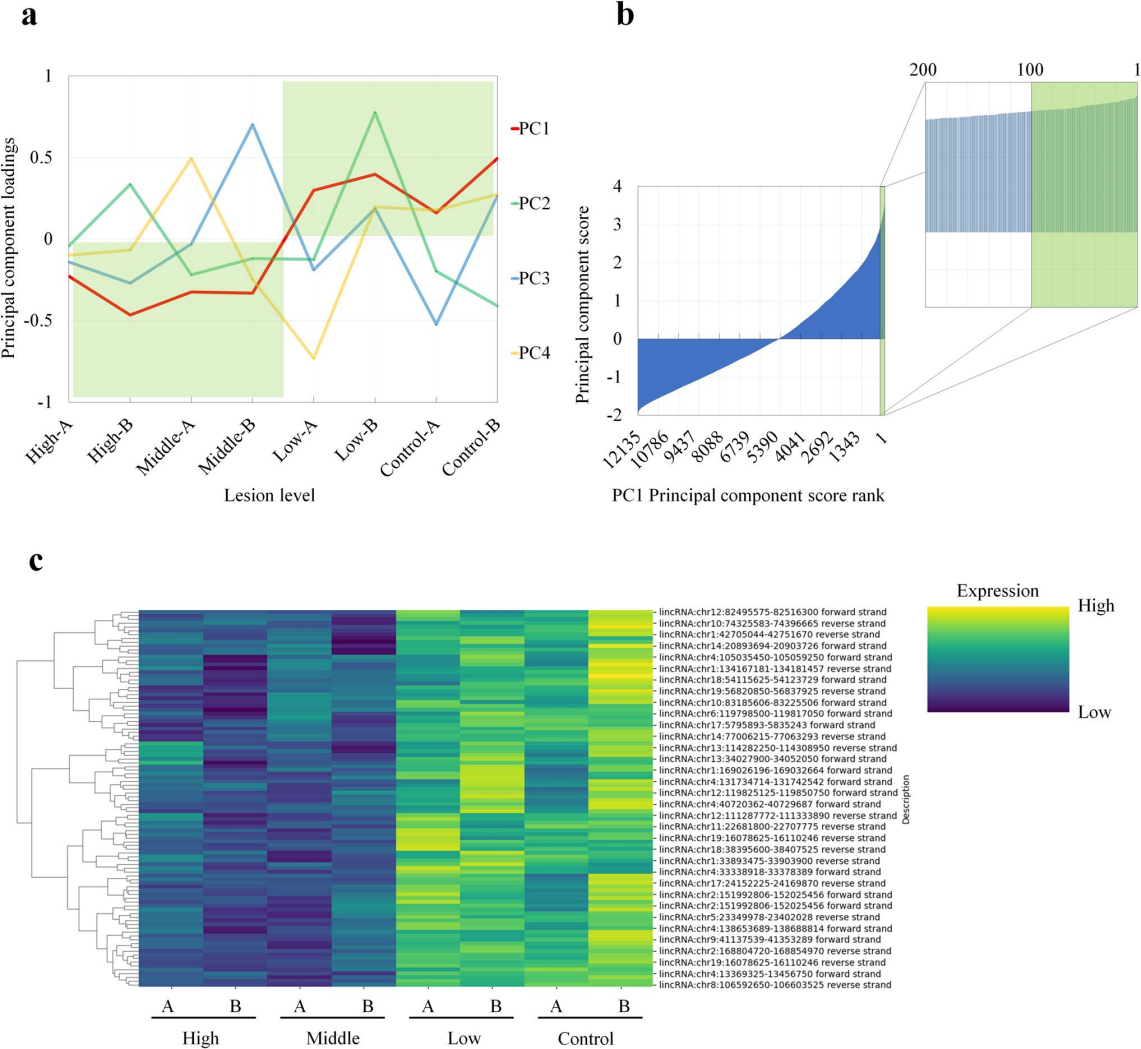
PCA for extracting lncRNAs downregulated in the lesion. **a** Principal component loadings. Lesion level (High-A, B: 2 samples with severe lesions, Middle-A, B: 2 samples with normal lesions, Low-A, B: 2 samples with weak lesions, Control-A, B: 2 samples in Control). **b** PC1 principal component score rank. The enlarged figure shows the portion of the principal component scores from the 1 st to the 200th ranks. The green area indicates the portion from the 1 st to the 100th place. **c** Heat map obtained by hierarchical clustering. In the heat map, higher expression intensity is indicated by darker yellow, and lower expression intensity is indicated by darker blue

To identify lncRNAs involved in the suppression of perivascular lesions in the brain, lncRNAs that correlated inversely with the extent of lesions were extracted using PCA. To eliminate arbitrariness and bias, we visualized the characteristics of the expression profiles (Fig. 1a) and extracted lncRNAs downregulated in the lesions in this study. The principal components (PCs), created by PCA, to be focused on were examined from the graphs of the contribution ratio and principal component addition amount (Table 1, Fig. 1a). PC1 was the principal component with the largest contribution, accounting for approximately 30% of the original data (Table 1). PC1 had negative principal component loadings in the two samples with severe lesions and the two samples with normal lesions and positive principal component loadings in the two samples with weak lesions and the two control samples (Fig. 1a). Therefore, PC1 was the principal component that significantly captured differences due to lesion effects and was suitable for extracting lncRNAs that were inversely correlated with the extent of lesions.

**Table 1:** Contribution rate

Principal component	Contribution rate
Principal component 1 (PC1)	0.228
Principal component 2 (PC2)	0.150
Principal component 3 (PC3)	0.143
Principal component 4 (PC4)	0.130

Hence, PC1 principal component information and scores were used to identify lncRNAs involved in the suppression of perivascular lesions in the brain (Fig. 1b). LncRNAs with the highest principal component scores ranked in the PC1 principal component scores were largely responsible for the positive principal component loadings in the two samples with the weakest degree of lesions and the two control samples. Therefore, lncRNAs ranked in the top 100 in principal component scores were extracted, and a heat map was created using hierarchical clustering to visualize the expression variation of the extracted lncRNAs (Fig. 1c). The 100 extracted lncRNAs were expressed at low levels in the two samples with the most severe lesions and the two samples with normal lesions and at high levels in the two samples with the weakest lesions and the two samples in the control group. Therefore, the lncRNAs extracted by principal component analysis were considered to be involved in the suppression of lesions, as their expression was inversely correlated with the degree of lesions.

### Alignment analysis with Clustal Omega

**Fig. 2:**
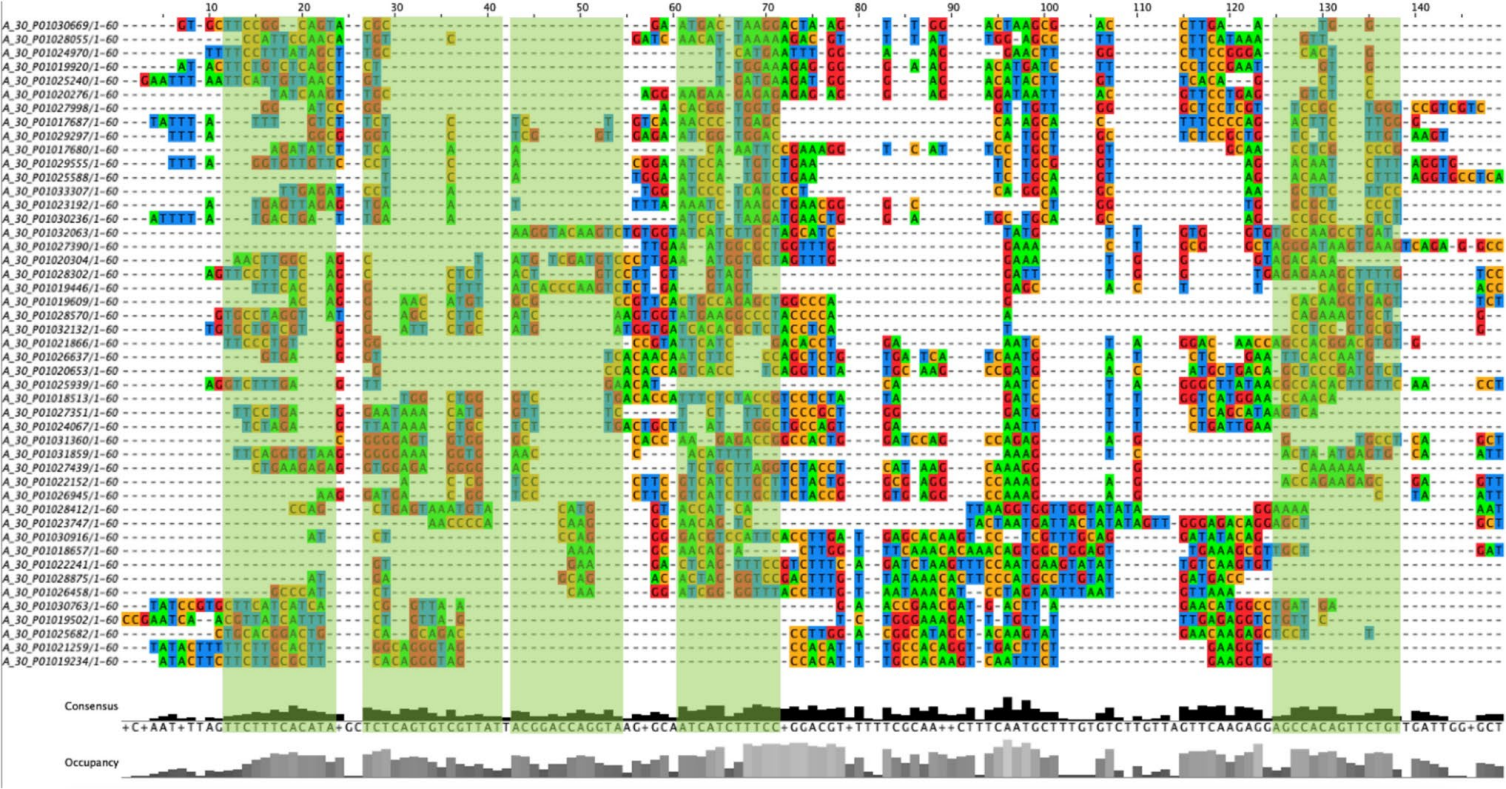
Selected probe sequence alignment results of the 100 lncRNAs extracted. The vertical direction indicates the probe ID of each lncRNA, and the horizontal direction indicates the probe sequence (A: green, G: red, T: blue, C: orange). Sequences that met the extraction conditions in consensus at the bottom of the alignment results were extracted as common sequences of lncRNAs. Although thymine (T) becomes uracil (U) in RNA sequences, it is represented as thymine based on the settings in the tool used for the analysis

Alignment analysis with Clustal Omega was performed to identify common sequences among the 100 lncRNA sequences extracted by PCA. We identified five sequences and their complementary sequences as common sequences among the extracted lncRNA sequences (Fig. 2, Table 2).

**Table 2:** Common sequences of lncRNAs extracted and their complementary sequences

Common sequences	Sequences	Number of bases	Complementary sequences
Common sequence 1	UUCUUUCACAUA	12	UAUGUGAAAGAA
Common sequence 2	UCUCAGUGUCGUUAU	15	AUAACGACACUGAGA
Common sequence 3	ACGGACCAGGUA	12	UACCUGGUCCGU
Common sequence 4	AUCAUCUUUCC	11	GGAAAGAUGAU
Common sequence 5	AGCCACAGUUCUGU	14	ACAGAACUGUGGCU

### Search for mRNAs with complementary sequences

The identified complementary sequences may be possessed by RNAs that likely regulate gene expression by complementary binding to each common sequence of lncRNAs in vivo. BLAST and the Human Protein Atlas were used to identify mRNAs that had complementary sequences and were expressed in the cerebral cortex. Finally, 415 mRNAs with at least one of the complementary sequences 1–5 and expressed in the cerebral cortex of the brain were extracted (Table 3).

**Table 3:** mRNAs that have complementary sequences and are expressed in the cerebral cortex

Complementary sequence	Number of mRNAs	Number of mRNAs expressed in the cerebral cortex	Confirmation of the existence of a function	Number of functions
Complementary sequence 1	101	87	Existence	2
Complementary sequence 2	4	3	No existence	-
Complementary sequence 3	15	14	No existence	-
Complementary sequence 4	321	285	Existence	22
Complementary sequence 5	35	32	No existence	-
Total	470 (476)	415 (421)		

Complementary sequences 1–5 are the complementary sequences of common sequence 1–5, respectively. Values in parentheses represent the total number of included mRNAs duplicated in complementary sequences 1–5. Existence indicates statistically significant functions that were extracted; no existence indicates that no statistically significant features were extracted. The number of functions is indicated as “-” if no statistically significant functions were extracted

### Statistical functional analysis of extracted mRNA groups using DAVID

**Fig. 3:**
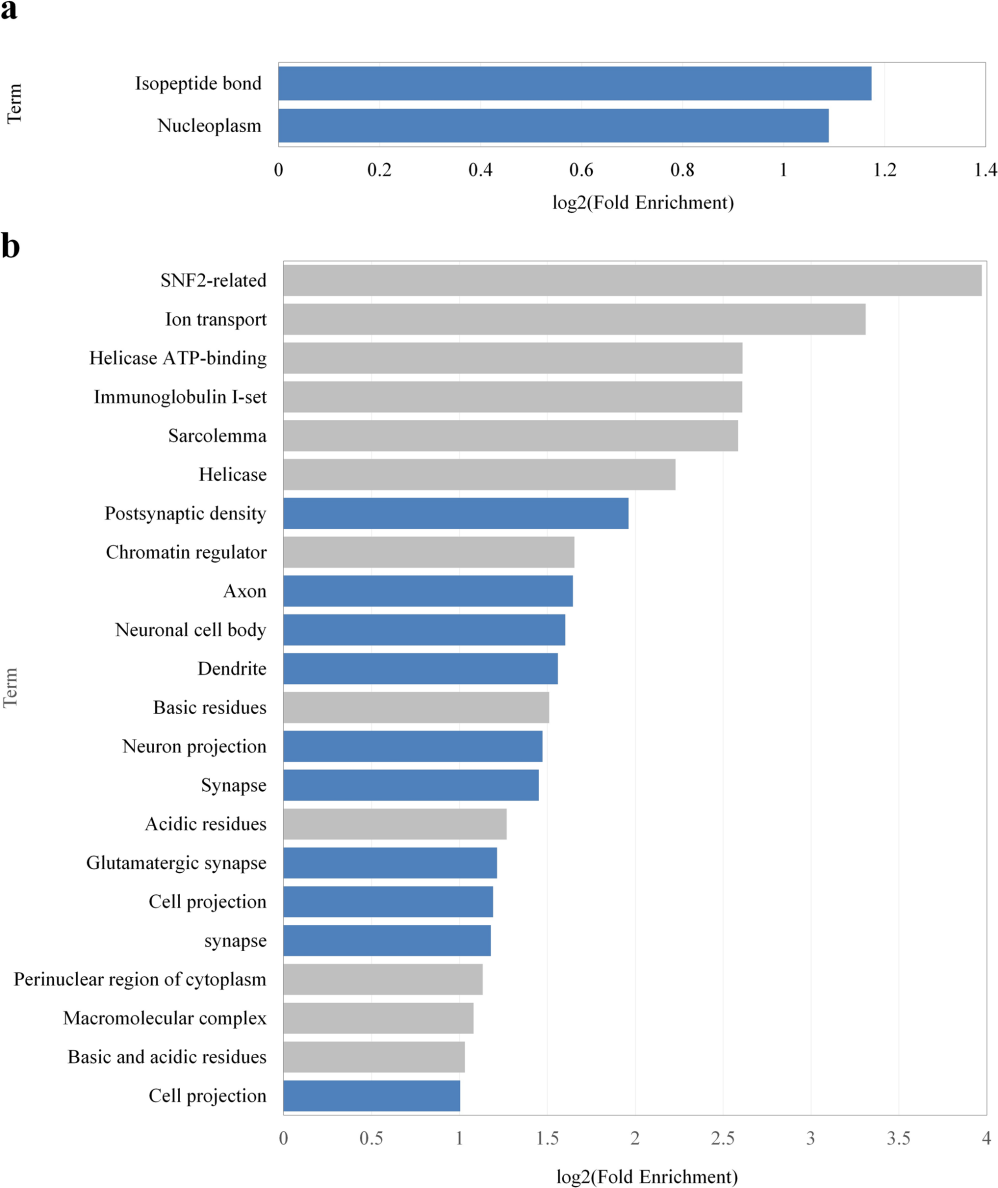
Functions enriched in the group of mRNAs with the complementary sequences. Functions identified to be statistically significantly enriched in the mRNA group with **a** complementary sequence 1 and **b** complementary sequence 4 are shown. Blue-shaded regions represent the functions of mRNAs expected to be involved in lesions

Statistical functional analysis was performed using DAVID for each group of mRNAs with complementary sequences. DAVID analysis revealed that the mRNA groups with statistically significant functions were mRNA groups with complementary sequences 1 and 4 (Table 3). The functions identified (using DAVID) to be statistically significantly enriched in the mRNA group with complementary sequence 1 were “Nucleoplasm” and “Isopeptide bond” (Table 4, Fig. 3a). The functions identified (using DAVID) to be statistically significantly enriched in the mRNA group with complementary sequence 4 were “Basic and acidic residues,” “Neuronal cell body,” “Postsynaptic density,” “Immunoglobulin I-set,” “SNF2-related,” “Cell projection”, “Dendrite,” “Basic residues,” “Axon,” “Sarcolemma,” “Acidic residues,” “Synapse”, “Ion transport,” “Neuron projection,” “Helicase ATP-binding,” “Macromolecular complex,” “Perinuclear region of cytoplasm,” “Helicase,” “Chromatin regulator,” “Glutamatergic synapse” (Table 5, Fig. 3b).

**Table 4:** Functions identified to be statistically significantly enriched in the group of mRNAs with complementary sequence 1

Category	Term	LH	LT	PH	PT	Fold enrichment	FDR
UP_KW_PTM	Isopeptide bond	19	70	1569	13,044	2.257	0.005
GOTERM_CC_DIRECT	Nucleoplasm	29	79	3674	21,301	2.128	0.012

*PT*, population total; *PH*, population hits; *LT*, list total; *LH*, list hits; *FDR*, false discovery rate

“Category” indicates the method used for data collection and “Term” indicates the function. PT indicates the total number of genes of the selected species obtained by the method indicated in Category. PH indicates the number of genes with the functions indicated in Term among the genes in PT. LT indicates the number of genes with the functions indicated in Term among the list of genes analyzed. LH indicates the number of genes in LT that possess the function indicated in Term. “Fold enrichment” included in the extraction conditions is the calculated value of (LH/LT)/(PH/PT), and FDR indicates the percentage of false positives among those for which the test results were determined to be significant but not actually significant

**Table 5:** Functions identified to be statistically significantly enriched in the group of mRNAs with complementary sequence 4

Category	Term	LH	LT	PH	PT	Fold enrichment	FDR
INTERPRO	SNF2-related	7	273	34	20,806	15.69	0.001
UP_SEQ_FEATURE	Ion transport	7	280	56	22,228	9.923	0.020
UP_SEQ_FEATURE	Helicase ATP-binding	9	280	117	22,228	6.107	0.028
INTERPRO	Immunoglobulin I-set	12	273	150	20,806	6.097	0.001
GOTERM_CC_DIRECT	Sarcolemma	11	277	141	21,301	5.999	0.001
UP_KW_MOLECULAR_FUNCTION	Helicase	9	168	139	12,161	4.687	0.027
GOTERM_CC_DIRECT	Postsynaptic density	19	277	375	21,301	3.896	0.000
UP_KW_MOLECULAR_FUNCTION	Chromatin regulator	13	168	299	12,161	3.147	0.027
GOTERM_CC_DIRECT	Axon	21	277	516	21,301	3.130	0.001
GOTERM_CC_DIRECT	Neuronal cell body	26	277	659	21,301	3.034	0.000
GOTERM_CC_DIRECT	Dendrite	24	277	626	21,301	2.948	0.001
UP_SEQ_FEATURE	Basic residues	24	280	669	22,228	2.848	0.006
GOTERM_CC_DIRECT	Neuron projection	21	277	582	21,301	2.775	0.004
UP_KW_CELLULAR_COMPONENT	Synapse	20	253	520	17,987	2.734	0.003
UP_SEQ_FEATURE	Acidic residues	29	280	956	22,228	2.408	0.010
GOTERM_CC_DIRECT	Glutamatergic synapse	20	277	663	21,301	2.320	0.040
GOTERM_CC_DIRECT	Cell projection	37	277	1246	21,301	2.284	0.001
GOTERM_CC_DIRECT	Synapse	30	277	1019	21,301	2.264	0.004
GOTERM_CC_DIRECT	Perinuclear region of cytoplasm	24	277	842	21,301	2.192	0.025
GOTERM_CC_DIRECT	Macromolecular complex	26	277	946	21,301	2.114	0.025
UP_SEQ_FEATURE	Basic and acidic residues	139	280	5402	22,228	2.043	0.000
UP_KW_CELLULAR_COMPONENT	Cell projection	34	253	1205	17,987	2.006	0.003

“Category” indicates the method used for data collection and “Term” indicates the function. PT indicates the total number of genes of the selected species obtained by the method indicated in Category. PH indicates the number of genes with the functions indicated in Term among the genes in PT. LT indicates the number of genes with the functions indicated in Term among the list of genes analyzed. LH indicates the number of genes in LT that possess the function indicated in Term. “Fold enrichment” included in the extraction conditions is the calculated value of (LH/LT)/(PH/PT), and FDR indicates the percentage of false positives among those for which the test results were determined to be significant but not actually significant

## Discussion

In this study, RNA expression profile data was provided for preprocessing including normalization and then subjected to the following analyses, enabling the extraction of lncRNAs that are differentially expressed in the target lesions with minimized bias from random individual variation. Low-expression lncRNAs were excluded during the data preprocessing to ensure reliable identification of lncRNAs. This approach is useful for identifying suppression targets in various pathological conditions. Although the number of transcriptome data samples used is limited, their reproducibility has been confirmed across multiple studies at both the transcriptional and protein levels in some genes with histopathological observations (Onoda et al. 2017b, 2017c; Umezawa et al. 2018). This study extracted sequence-based potential functions of lncRNAs that are downregulated in the target perivascular lesion in the brain. Functional enrichment analysis of mRNAs that can bind to the extracted lncRNAs via complementary sequences suggested that the mRNA group may be involved in the pathogenesis of the lesion. Statistical functional analysis using DAVID extracted a total of two functions, “nucleoplasm” and “isopeptide binding,” that are statistically significantly enriched in the mRNA group with complementary sequence 1. “Nucleoplasm” refers to the liquid portion of the nucleus enclosed by the nuclear membrane and contains several non-membrane-bound structures, such as nucleoli and nuclear speckles. Its primary function is to store DNA and enable DNA-dependent processes such as transcription in a controlled environment. Disruption in nucleoplasmic processes can lead to defective transcriptional regulation, which may exacerbate lesion pathology. Therefore, problems with gene regulatory processes in the nucleoplasm may be related to the production and processing of abnormally structured proteins (Galganski et al. 2017). “Isopeptide bond” is an amide bond between the C-terminal carbonyl group of ubiquitin and the lysine ε-amino group during ubiquitin modification. Neurodegenerative diseases associated with perivascular lesions in the brain develop when abnormally structured proteins accumulate intracellularly or extracellularly in neurons and other normal cellular structures, resulting in structural and functional degeneration of the central and peripheral nervous systems (Fang et al. 2022). Since isopeptide bonds formed by transglutaminase are stable and difficult to degrade, pathological proteins such as amyloid-β and α-synuclein irreversibly bind to each other if excess isopeptide bond is formed, leading to the formation of abnormal protein aggregates (Zhang et al. 2016). The ubiquitin-proteasome pathway is a proteolytic pathway through which eukaryotic cells process abnormally structured or damaged proteins (Ciechanover 2005). Therefore, isopeptide binding related to the ubiquitin-proteasome pathway may be a relevant function in perivascular lesions in the brain and neurodegenerative diseases.

A total of 22 functions were extracted as statistically significantly enriched in the mRNA group with complementary sequence 4. Astrocyte activation, characterized by Gfap upregulation, is a hallmark of perivascular lesions in the brain. Astrocytes are glial cells that fill the brain, and their functions include housekeeping functions necessary to maintain neuronal function and to actively form synapses via synthesizing extracellular matrix proteins, adhesion molecules, and trophic factors (Wang and Bordey 2008). Housekeeping functions include buffering excess potassium and neurotransmitters, providing perisynaptic nutrients and structural support, and contributing to the integrity of the blood–brain barrier (Wang and Bordey 2008). In addition to their function in actively forming synapses, astrocytes are involved in the release and uptake of various neurotransmitters, with an emphasis on neurons at the synapses and their removal (Chung et al. 2015). Also, astrocytes regulate glutamatergic synaptic function through glutamate reuptake and signaling via NMDA receptors (Chipman et al. 2021). The focused lesion in the present study, persistent astrocytic activation with Aqp4 overexpression in the brain perivascular areas, is related to impaired clearance of waste products such as abnormally folded proteins (Onoda et al. 2017a, 2017c). Among the identified 22 functions enriched in the potential target mRNAs for the lncRNAs downregulated in the lesion, “Neuronal cell body,” “Synapse,” “Postsynaptic density,” “Cell projection,” “Dendrite,” “Neuron projection,” and “Glutamatergic synapse” are functions related to astrocytes.

Alzheimer's disease, a typical example of neurodegenerative disease, is characterized by senile plaques composed of amyloid-β in the patient’s brain, neurofibrillary changes composed of intracellular hyperphosphorylated microtubule-associated protein tau, dystrophic neurites, decreased synaptic density, and neuronal loss. Thus, these eight functions may be related to decreased synaptic density and neuronal loss and perivascular lesions and neurodegenerative diseases.

The identified functions, based on complementary sequences 1 or 4, are related to perivascular lesions in the brain and neurodegenerative diseases. However, among the two functions associated with complementary sequence 1, the range of functions for “nucleoplasm” is broad, and its direct involvement in perivascular lesions in the brain is difficult to verify. In contrast, with respect to functions that were associated with complementary sequence 4, eight functions can be linked to perivascular lesions in the brain. In addition, the group of mRNAs with complementary sequence 4 contained *Gfap*, a molecule important for astrocyte and blood-brain barrier functions and implicated in the pathogenesis of brain perivascular lesions (Onoda et al. 2017c). Therefore, there is a possibility that “AUCAUCUUUUCC” of common sequence 4 and *Gfap* bind complementarily and regulate the expression of Gfap by the principle of lncRNA trans-acting (Yoon et al. 2013), and regulate the astrocyte differentiation induction and cytoskeleton maintenance functions of Gfap.

Thus, among the common sequences 1–5 of the extracted lncRNAs, the sequence with the greatest potential to suppress perivascular lesions in the brain is the common sequence 4, “AUCAUCUUUCC.” Therefore, lncRNAs with this sequence (derived from the consensus sequence 4) may control the expression and functioning of mRNAs with statistically significantly enriched functions by binding complementarily.

In this study, lncRNAs involved in the suppression of perivascular lesions in the brain were defined as those whose expression correlated inversely with the extent of perivascular lesions in the brain; however, it should be noted that RNAs that correlated inversely with lesions do not always contribute to the suppression of perivascular lesions in the brain. In addition, we focused on sequences common to a group of lncRNAs whose expression correlated inversely with the degree of perivascular lesions in the brain. However, it is possible that one of the individual lncRNAs is important instead of the common sequence, and it would be meaningful to examine this possibility. Additionally, even if the pathology of the perivascular astrogliosis has a similarity to the neurodegenerative and neurodevelopmental disorders with abnormal neurobehavior (Umezawa et al. 2018), the effects of lncRNAs on brain diseases are unsolved issues that require more investigations. Further experimental validation is needed to examine whether the lncRNAs and their common sequences extracted in this study suppress perivascular lesions and potentially related brain diseases.

## Conclusion

In this study, we extracted lncRNAs that were dysregulated by the development of perivascular lesions in the cerebral cortex induced by exposure to carbon black nanoparticles during the fetal period. Common sequences are present in the lncRNAs whose expression decreases in brain perivascular lesion. A common sequence “AUCAUCUUUCC” was found in the lncRNAs whose expression inversely correlated with the level of the perivascular lesions in the brain. As a result of the prediction of the function of this sequence by identifying in mRNAs that can bind, eight GO terms, including “Cell projection” and “Dendrite,” were extracted as the functions of mRNAs possessing sequences complementary to the identified lncRNAs and potentially involved in suppressing the lesions. These functions were closely related to the pathology of brain perivascular lesions, such as neural dysfunction and astrocytic endfoot elongation. lncRNAs with this sequence may represent novel molecules that suppress brain perivascular astrogliosis, which is linked to neurodegenerative and neurodevelopmental disorders.

## Data Availability

The data and codes that support the findings of this study are available from the corresponding author (MU), upon reasonable request.

## Abbreviations

Aqp4: Aquaporin 4
BLAST: Basic Local Alignment Search Tool
DAVID: Database for Annotation, Visualization, and Integrated Discovery
Gfap: Glial fibrillary acidic protein
lncRNA: Long non-coding RNA
miRNA: MicroRNAs
PCA: Principal component analysis
